# No learning where to go without first knowing where you're coming from: action discovery is trajectory, not endpoint based

**DOI:** 10.3389/fpsyg.2013.00638

**Published:** 2013-09-18

**Authors:** Martin Thirkettle, Thomas Walton, Peter Redgrave, Kevin Gurney, Tom Stafford

**Affiliations:** ^1^Department of Psychology, The Open UniversityBuckinghamshire, UK; ^2^Department of Psychology, University of SheffieldSheffield, UK

**Keywords:** action outcome learning, spatial learning, intrinsic motivation, cognitive science, motor learning

## Abstract

Intrinsic motivations drive an agent to explore, providing essential data for linking behaviors with novel outcomes and so laying the foundation for future flexible action. We present experiments using a new behavioral task which allows us to interrogate the connection between exploration and action learning. Human participants used a joystick to search repeatedly for a target location, only receiving feedback on successful discovery. Feedback delay was manipulated, as was the starting position. Experiment 1 employed stable starting positions, so the task could be learnt with respect to a target location or a target trajectory. Participants were able to learn the correct movement under all delay conditions. Experiment 2 used a variable starting location, so the correct movement could only be learnt in terms of target location. Participants displayed little to no learning in this experiment. These results suggest that movements on this scale are stored as trajectories rather than in terms of target location. Overall the experiments demonstrate the potential of this task for uncovering the native representational substrates of action learning.

## Introduction

When Thorndike (1911) pioneered the study of action acquisition with his puzzle box escape paradigm, he was investigating whether animals can learn to produce apparently insightful behavior despite having no causal understanding of the problem at hand. By repeatedly placing subjects into a puzzle box and measuring the time taken for them to enact their escape, Thorndike was able to observe and record the animals as they gradually extracted the elements of behavior associated with success from a complex stream of self-generated behavioral variance. Thorndike's animals improved across trials, and while they may have had little insight into the underlying relationship between their actions and escape, the feat of learning was nonetheless impressive, requiring them to solve a considerable problem of credit assignment (Minsky, 1961; Sutton and Barto, 1998). The major challenge of learning through trial and error is that success will inevitably be associated with both causally relevant and causally irrelevant activities. In addition to this, the learning system must deal with delays between successful actions and their associated outcomes—the so called “distal reward problem” (Izhikevich, 2007), with no way of determining how far back in the motor record the most important aspects of performance might lie.

By associating motor activity with a particular outcome, animals create an action-outcome pair, which can then be added to the behavioral repertoire. Theories of the representation of action suggest that it is the outcome which is represented after learning (Hommel et al., 2001), but these focus on the goal of the action, rather than how to perform the action. During action discovery, especially in a situation where discovery occurs during unconstrained exploration, the contingency is not necessarily obvious, and moreover identifying the causal element of motor output is non-trivial. In normative models of reinforcement learning, a common method is to use temporal difference algorithms which maintain a trace of the pattern of recent activity, such that it remains eligible for reinforcement at the moment when the outcome eventually occurs (Barto et al., 1981; Wickens, 1990; Singh and Sutton, 1996). This approach is consistent with Skinner's studies of superstitious behavior (1948), and predicts that participants will learn sub-optimal strategies based on prior success, as previously successful trajectories of movement will be reinforced regardless of the underlying contingency of the action-outcome pair. It is also clear that this mechanism of associating recent motor activity with success leaves little opportunity for insight into which aspect of the previously successful movement is causal and which can be pruned across repetitions. Such refinements could only occur through a process of trial-and-error across numerous action repetitions.

Redgrave and Gurney (2006) and Redgrave et al. (2008) have argued that the response of dopamine neurons in the ventral midbrain ~100 ms after the presentation of novel and rewarding stimuli acts as an indiscriminate time-stamp which indicates the last segment of the animal's motor record that could have played a role in eliciting a novel stimulus, irrespective of what that stimulus might be. They propose that the dopamine response is central to the tasks of agency detection, action discovery and the learning of action-effect contingencies. It is widely thought that this activity plays a key role in valuation and economic decision-making (Schultz et al., 1997; Schultz, 2007), and in the case of action discovery, Redgrave and Gurney (2006) suggest that the dopamine response acts as the timestamp against which the motor commands in the eligibility trace can be compared—ameliorating the distal reward problem. While this time-stamping mechanism prevents any motor commands subsequent to the outcome, and therefore non-contingent, from entering the pool of potential contingencies, the record of recent motor output eligible for reinforcement will still contain non-contingent elements, and any manipulation of the time between movement performance and success being signaled will necessarily introduce further non-contingent motor output.

The twin problems of credit assignment and distal reward are at the heart of a paradigm we created to investigate this kind of “Thorndikian” action acquisition in human and animal participants (Stafford et al., 2012). This task captures the discovery of a novel action through self-generated behavior and allows the refinement of that behavior through the trial-and-error pruning of non-contingent elements to be studied. In this task participants move a joystick freely and “escape” the trial by discovering the action set by the experimenter, in this case simply placing the joystick controlled cursor within a pre-defined area. The participants receive no feedback on the cursor's location and successful performance of this action is denoted only by an audio signal and the end of the trial. Other work using this paradigm has focused on the neural pathways preferred for processing the reinforcing signal (Thirkettle et al., 2013), or on the time sensitivity of these mechanisms (Walton et al., 2013). Together this work seeks to better understand the cognitive mechanisms and neural pathways involved in the discovery and learning of novel actions through self-generated exploratory behaviors. Stafford et al. (2012) include an in depth discussion of the nature of the joystick task and its relationship to previous behavioral work studying learning. The present experiments aim to identify if an “eligibility trace” of movement trajectories generated during an iterated location finding task is necessary for learning. Previous studies have focused on the discovery of a new action-outcome pair; here that moment of discovery is studied alongside the refinement of the action through repetition. If participants learn a novel action by stamping in recent motor output, there should be evidence of this in the form of the preservation of portions of successful movements from early performances in later ones. The design of the joystick task, lacking as it does, any visual information regarding either the target location, or the current location of the joystick in the search arena encourages the participants to use motoric and bodily sources of information. The type of location information used in a location finding task has been shown to affect performance in terms of both systematic biases and absolute levels of performance (e.g., Simmering et al., 2008), but here our focus is on maintaining a constant source of information—proprioception and efference copy—and manipulating the usefulness of relevance of past experience to inform learning. If a reliance on the movements made previously is found, we would predict this would preclude learning in a situation where only the endpoint of a previous movement, rather than the movement itself was informative.

We therefore sought to measure learning performance when the eligibility trace was contaminated with additional, non-contingent, motor commands, and when the record of motor commands was devalued across movement repetitions. In experiment one, participants discovered the location of a hidden target area and then repeated moving to this target from the same start position. By manipulating the delay between the participant entering the target area and the presentation of the success signal, contamination was introduced into the record of recent motor commands. If the eligibility trace is bound by a time-stamping mechanism then increasing this delay between action performance and reinforcement should produce weaker learning and more variable movement trajectories across repetitions. In experiment two, we repeated the manipulation of delay in the location finding task but used a randomized starting location for each repetition of the movement to the target—forcing participants to return to the target area from a different position each time. If participants are relying on the previous movements rather a representation of the target location to refine their performance across repetitions then both learning and performance should be extremely poor.

## Experiment 1

### Materials and methods

#### Participants

Thirty undergraduate students (mean age 19 years) at the University of Sheffield (25 females) participated in all conditions of this study. Participants took part in return for credits in the department's research participation scheme. All subjects were naive to the purpose of the experiment and the independent variable. Ethical approval was granted by the department's ethics committee.

#### Apparatus

The experimental program was written in Matlab (Version 2007), and stimulus display was performed using the Psychophysics Toolbox extensions (Brainard, 1997). A 19″ monitor was used throughout along with a standard USB keyboard for participant response during instructions. A commercial joystick (Logitech extreme 3D pro joystick, P/N: 863225-1000) was used as the experimental input device. Custom Matlab code polled the position of the joystick at 100 Hz.

The search space was defined as a square with a side length of 1024 units. Movements of the joystick were mapped onto movements within the search space in a one to one fashion, with the joystick starting in the center of the search space at the beginning of each trial. Once released from the grip of a participant, the joystick's internal spring returned it to the center of the search space within a tolerance of 10 units.

#### Procedure

Participants sat at a desk in front of the joystick and monitor. Before starting the experimental program, the task was briefly described verbally with the goal being phrased as “finding the correct position to place the joystick in.” Participants were told that the experiment involved no deception and that the correct position could always be found. Following this brief verbal reassurance, the program was started and the participants were asked to follow the onscreen instructions.

The size of the target area (hotspot) that participants were required to find was determined through pilot testing and set to occupy 0.28% of the search space. Experimental trials were split into ten iterations, an initial iteration where they had to search for the hotspot, and nine subsequent iterations where they had to return to a hotspot in the same position. Each iteration began with the joystick in the center of the search space and for each trial the center of the hotspot was positioned randomly within an annulus shaped region of the total search space to ensure that the hotspot never overlapped the central starting point or the outer edge of the search space. During an iteration any movement of the joystick into this region was defined as a “hit” and resulted in a beep (600 Hz) of 10 ms duration and the end of the iteration. During each iteration, the screen remained dark and blank. A delay period of 0, 150, 300, or 450 ms was interposed between the moment at which a participant moved into the hotspot and the point at which the beep was presented. This also marked the end of the current iteration and was accompanied by an on-screen message to prepare for the next (see Appendix 1 for full details of onscreen text).

Before the experimental trials, participants completed a short practice session and once this was completed the experimental trials began immediately. Participants completed 2 trials at each delay duration, each trial containing 10 iterations—for a total of 80 movements. Order of trial delay condition was counterbalanced across participants to control for order effects.

#### Data analysis

We used a 4th order two-way low pass Butterworth filter at 10 Hz to remove noise and redundant data points from the movement data. This filter is commonly used in studies of human motion (Seidler, 2007) and smoothed the raw joystick output, the intention being to more accurately reflect the underlying movement of the participant's hand and arm.

**Figure 1:**
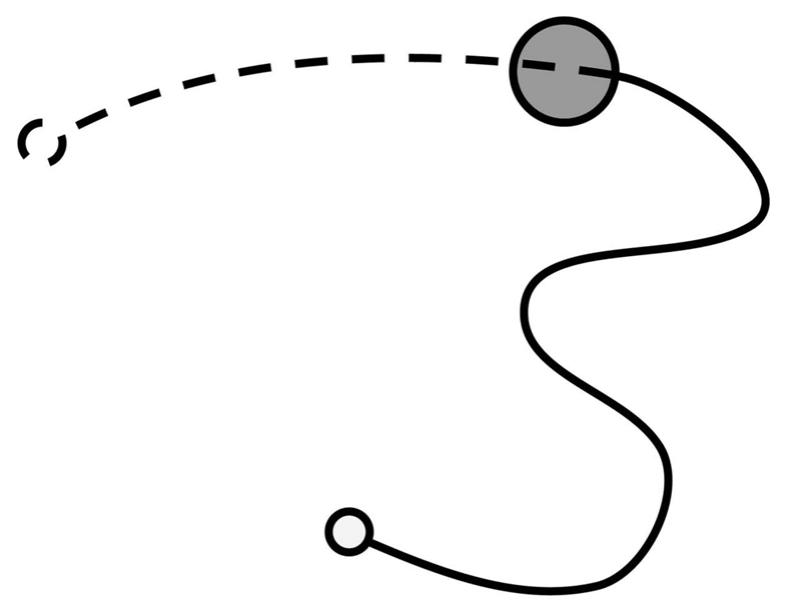
**Illustration of joystick movement trace showing phases of joystick movement: The movement trace was split into the activity between the start of the iteration (solid, unfilled, circle) and encountering the target (filled circle), and the activity between encountering the target and the presentation of the signal (dashed, unfilled, circle).** These phases of movement were termed the pre-discovery (solid line) and post-discovery (dotted line) components.

For the purposes of analysis, the trace of movement from each iteration was treated as being composed of two phases: pre-discovery and post-discovery (Figure 1). The pre-discovery period extends from the start of the iteration to the point of entry into the hotspot and is free to last as long as the participant takes to discover the target. Conversely, the duration of the post-discovery period is strictly dictated by the delay imposed by the experimenter between the successful discovery of the target and the presentation of success signal. The post-discovery period is of particular interest as it contains contaminating—non-contingent—information produced by the participant. If people learn by stamping in recent motor output, their activity during this period should influence their performance during the pre-discovery period of subsequent trials.

Due to the open-ended nature of trials, it was anticipated prior to testing that the distribution of trial duration and distance covered would be positively skewed. Analysis of the data distributions confirmed this and all data were corrected using log-transformation prior to analysis (Keene, 1995).

### Results

In each iteration, the movement within the pre-discovery period can be compared against a direct line from the start position to the target. We term the difference between this straight line and the path taken by the participant the “irrelevant distance” and by collapsing this measure across the ten iterations of each trial, across trials and across participants the impact of the imposed signal delays on performance before contact was made with the target can be assessed.

**Figure 2:**
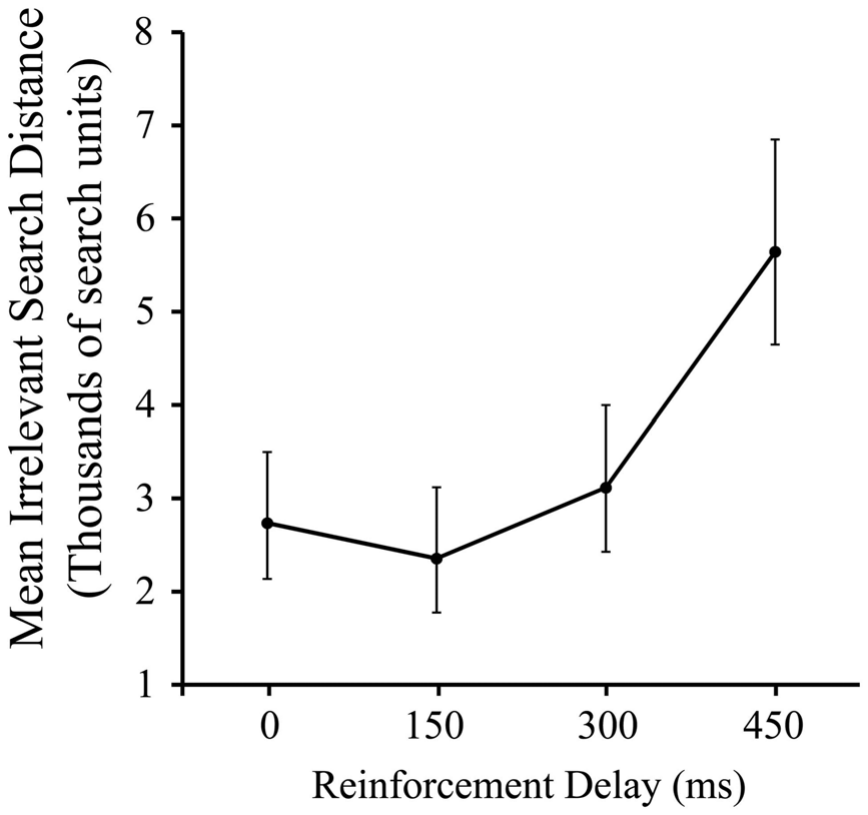
**Mean irrelevant pre-discovery distance (and standard error) for the 4 levels of reinforcement delay.** Values are back-transformed from the log transformation.

As Figure 2 shows, there was a significant effect of delay on pre-discovery irrelevant distance, *F*_(3, 87)_ = 4.79, *p* < 0.005, driven entirely by the slump in performance in the 450 ms condition (*p* < 0.05). This is a consistent, albeit less dramatic, effect to that reported in our previous work using a manipulation of signal delay in the joystick task, and we attribute the reduced delay sensitivity found here compared to the previous work to the iterated nature of the present task.

**Figure 3:**
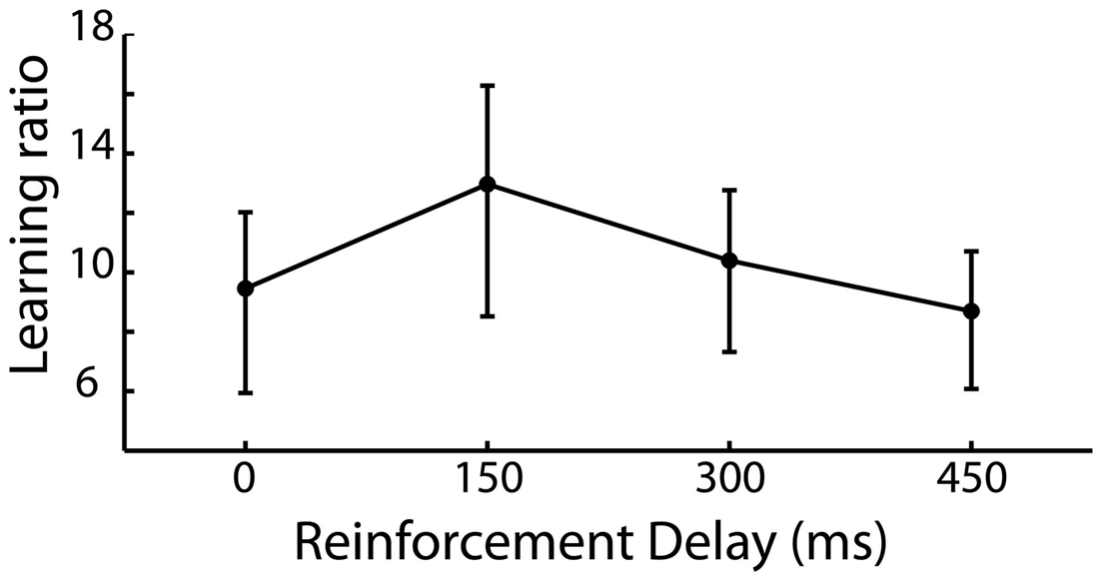
**Learning ratio (performance measures in the first half of iterations divided by that in the second half of iterations) for the 4 levels of reinforcement delay.** Values are back-transformed from the log transformation, error bars are standard error.

The requirement of the participant to repeat a newly acquired action allows the learning of that action to be captured and as such we expected to see a reduction in the irrelevant distance moved by the participant in later iterations of trials compared to earlier iterations of the same trial. A learning ratio was calculated by dividing the irrelevant distance traveled in the pre-discovery period in iterations 1–5 by that traveled in iterations 6–10 of each trial. Figure 3 shows the learning ratio collapsed across participants for the four delay conditions and shows that while there was no significant effect of delay on learning [*F*_(3, 87)_ = 0.142, *p* = 0.935], a considerable improvement in performance was observed from early to late trials. This suggests that the effect of delay on is one which impacts action discovery, rather than the refinement of the discovered action across the later iterations.

Within each trial, the refinement of the newly discovered action across iteration can be approximated by the power law of learning (Ritter and Schooler, 2001) as in the equation:

$$\text{efficiency}=\text{Em + range }\times \;e^{-\alpha N}$$

**Figure 4:**
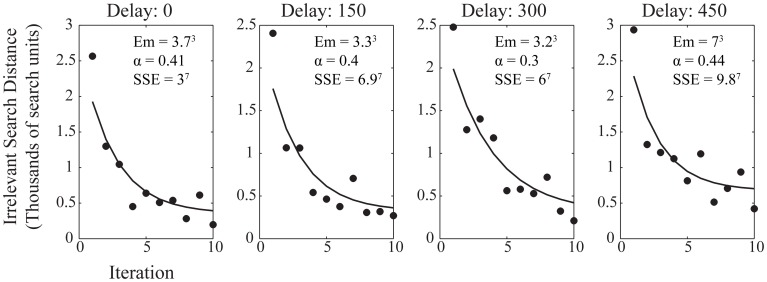
**Average participant performance across the 10 iterations for each delay condition.** Data shows the fitted power law curve which describes the rate of improvement in performance well. Annotations show the fitting values for the power law, both for the fitted variables of the power law itself and the sum of squares due to error (SSE) goodness of fit value for the curve fitted to the data.

Here we use irrelevant search distance as the measure of efficiency, with α being the parameter which describes the speed of learning with the range of observed performance levels, Em is a minimum figure for the irrelevant search distance, *range* is the difference between initial and asymptotic performance, and *N* is the number of trials. Figure 4 shows the average performance of participants within each trial at each reinforcement delay condition with the best power law fit applied. The improvement in performance is well-described by the power law at each level of delay, although, again, it is notable that the greater delay had more of an impact on the minimum irrelevant search distance than upon the value of α which describes the rate at which performance improved to asymptote. The similarity of α across delay conditions, as with the learning ratio, suggests that delay is impeding action discovery, rather than action refinement.

The learning evident in experiment 1, shows that after discovering the invisible target location in the first iteration, the movement required by the task was refined across the subsequent 9 iterations. Participants were able to greatly reduce the length of their path to the target by the final iteration compared to that taken on their first encounter with the target.

## Experiment 2

A limitation of using a stable starting position when seeking to investigate whether a particular trajectory of movement is stamped into behavior is that if participants perform close to optimally in an early trial, it is difficult to determine on subsequent trials whether similar trajectories of movement are a reflection of the participants adopting previously successful trajectories of movement or whether they are adopting previously successful trajectories simply because these trajectories are consistent with near optimal performance and they would have learnt to perform at this level anyway. An alternative approach is to vary the start position whilst maintaining a stable target. In this way it is possible to ensure that the optimal trajectory of movement varies from trial to trial, making it easier to determine whether participants are exploiting their memory of a previously successful movement path or learning a successful end point which they are able to reach from any starting position. Experiment 2 therefore replicated experiment 1, but after discovering the target location instead of repeating the movement from the same start position to the target 9 times, participants moved to the target from 9 randomly chosen start positions.

### Materials and methods

#### Participants

15 undergraduate students (mean age 19 years) at the University of Sheffield (11 females) participated in all conditions of this study. Again, participants took part in return for credits in the department's research participation scheme. All subjects were naive to the purpose of the experiment and the independent variable.

#### Apparatus

The experimental setup remained as in experiment 1; with the exception that stimulus display was performed using the Cambridge Research Systems Visage graphics board and the associated Matlab toolbox extensions. A Mitsubishi Diamond Pro 2070sb 22″ monitor was used throughout and a chin rest ensured the participants remained seated 57 cm from the screen throughout. Changes to the experimental code meant that the position of the joystick was now polled at 1000 Hz and the search space was defined as a square with a side length of 1000 units.

#### Procedure

All experimental procedures were kept as similar as possible as in experiment 1, with the addition of a requirement that the participant moved the joystick to a randomly selected start position before beginning the search on each iteration for the target. Also three rather than four different delay levels were employed in order to reduce experiment time, and focus more tightly on the most influential delays between success and reinforcement signal presentation. The randomization of start location was achieved by presenting the start position and the current position of the joystick on-screen and instructing the participant to move the cursor to the highlighted area in order to start the iteration. The start position was chosen in the same way as the target position (which, as in experiment 1, remained unchanged for the 10 iterations of each trial), with the additional constraint that it could not overlap the target position. As in experiment 1 participants understood that the target position was changing only for each trial of 10 iterations.

Participants again completed a short practice session immediately before the experimental trials and conducted three trials of 10 iterations at each of three delay levels (0, 200, and 400 ms) for a total of 90 trials. The resulting data was processed in the same way as in experiment 1 to correct for positive skew, and reduce redundant data points from the movement data.

### Results

**Figure 5:**
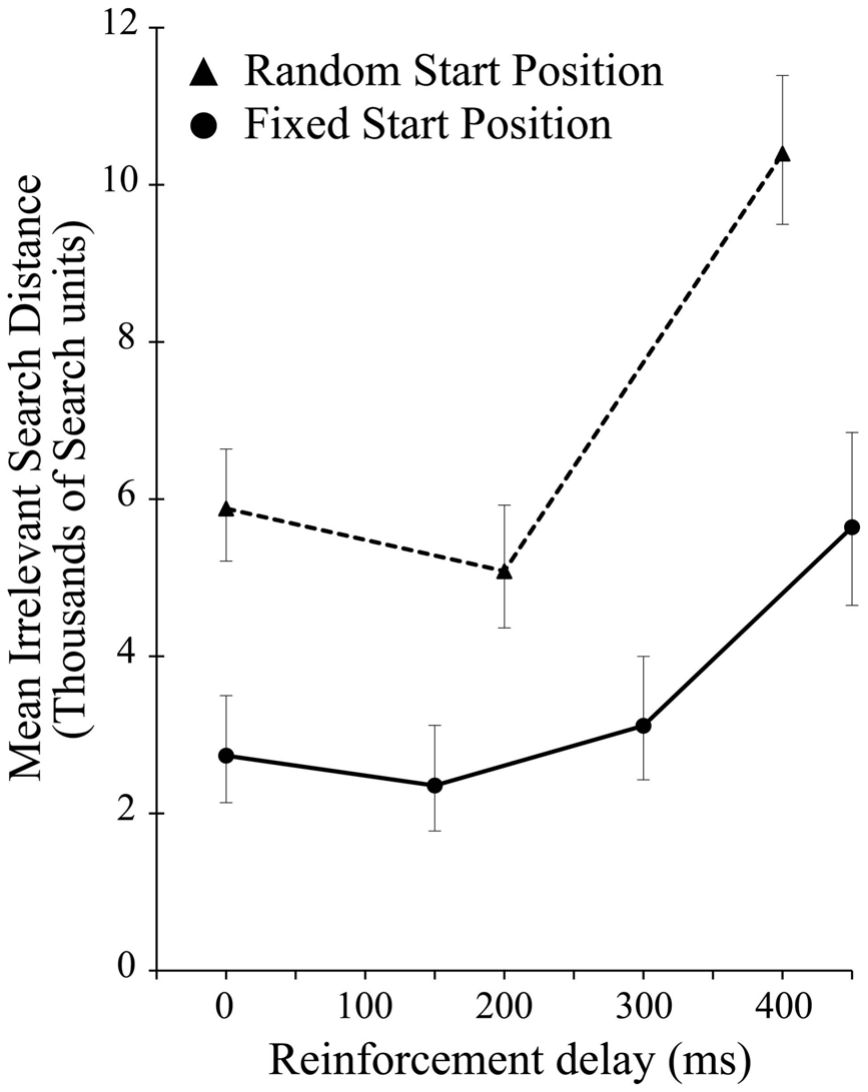
**Mean irrelevant pre-discovery distance (and standard error) for the 3 levels of reinforcement delay in experiment 2 (shown as dotted line).** The results from experiment 1 (solid line) are also plotted for comparison.

There was a significant effect of delay on the irrelevant distance traveled by the participants when the start position was randomized [*F*_(2, 28)_ = 13.422, *p* < 0.001]. As in experiment 1, this effect was driven entirely by the highest level of delay, in this case 400 ms [*F*_(1, 14)_ = 16.290, *p* = 0.001]. As Figure 5 shows, across all delay conditions, comparing between the experiments participants average irrelevant distance was greater when seeking a static target if the start position was changed from iteration to iteration suggesting a reliance on the static start position in order to find the unchanging target. While the delay manipulation is unequal across the two experiments, preventing in depth analysis, comparing performance in just the zero delay conditions, shows that changing the start position significantly impaired performance [*t*_(40.135)_ 2.709, *p* = 0.01].

**Figure 6:**
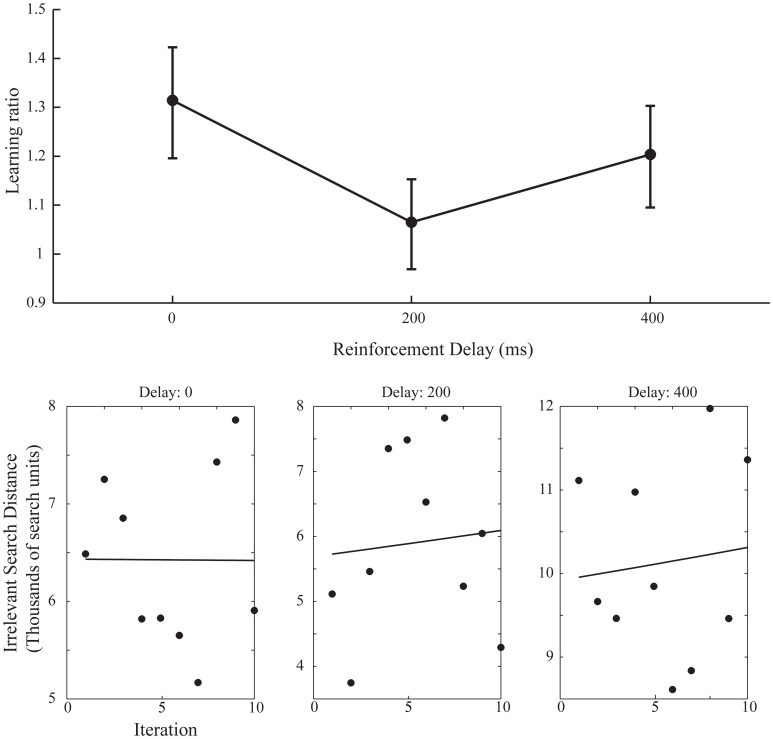
**Learning ratios and fitted performance data for experiment 2.** Compared to the learning ratios of experiment 1 (Figure 3) we again see no effect of delay on learning ratio but dramatically reduced values for said ratios (upper axis). Unlike in experiment 1 (Figure 4) the power law curve shown on the lower set of axis no longer adequately fits the data and participants did not reliably improve across the 10 iterations (Power law curve fit values for lower axis figures: 0 ms Delay: Em = 3.7^3^, α = 5.2^−4^, SSE = 7.1^6^, 200 ms Delay: Em = 1.6^3^, α = −9.4^−3^, SSE = 1.7^7^, 400 ms Delay: Em = 6.6^3^, α = −1.1^−2^, SSE = 1.2^7^).

A simple increase in the irrelevant distance traveled could signify that by changing the start position on each iteration the task of finding the target was made more difficult, rather than speaking to the effect of the changing start position on learning. However, this is revealed in the measures of learning across the ten iteration of experiment 2. Figure 6 shows the learning ratio and fitted power law data for performance in experiment 2. Again, we see no significant effect of delay on the learning ratio but the lack of improvement across the 10 iterations is striking. Unlike in experiment 1, participants did not improve as the repetitions of the movement continued, and this is borne out in a significant reduction in the learning ratio. Comparing the zero delay conditions across the two experiments again we see this reduction is significant [*t*_(35.724)_ 5.776, *p* < 0.01]. The lack of learning found without a stable start position is evidenced further by our attempts to fit a power law to the data as in experiment 1. The power law of learning no longer describes the data as no improvement in performance of any note is taking place. This strongly suggests that the refinement of the newly discovered action as found in experiment 1 is heavily reliant on a stable start position.

## General discussion

Whether the starting position was static (experiment 1) or changed with each iteration (experiment 2) participants were able to successfully discover the target location, but are affected by delaying the reinforcement signal. This is consistent with our previous findings using different versions of this task (Stafford et al., 2012; Thirkettle et al., 2013; Walton et al., 2013), but here we demonstrate the impact of reinforcement delay in a version of the task in which the reinforcement is delivered without giving the participant the opportunity to correct for, or respond to, the delay within a single performance of the reinforced action. Here participants had to repeat the entire action after a single, possibly delayed, reinforcement signal. This sensitivity to delay reveals, we argue that it is the process of action acquisition which is critically dependent on the coincidence of motor efference copy with a sensory signal indicating a novel or surprising outcome (Redgrave and Gurney, 2006) rather than the subsequent refinement of a discovered action through, in this case, repeated encounters with the target area. For the initial discovery of an action, the number of potentially causative movements grows with each moment and this record inevitably becomes increasingly contaminated with noise (i.e., movements or aspects of movement with no causative relationship to the action). Because of this, delivery of the sensory signal to the brain area(s) where it can be used to tag potentially causal elements in the motor record must be done as fast as possible in order to reduce the difficulty of the credit assignment problem (Minsky, 1961). In machine learning, the idea of an “eligibility trace” has been suggested as a mechanism for solving the credit assignment problem (Singh and Sutton, 1996). With regard to the joystick task, a system employing such an “eligibility trace” should display the repetition of aspects of movement contained within such a period regardless of their necessity for success. Further studies are planned to focus on the production and persistence of these “superstitious movements” in the joystick task.

Learning to move to a spatial target is significantly poorer, indeed, almost abolished, when only the target location remains static and the participant must move to the target without reference to their previous movements (experiment 2). This allows an additional supposition about how the credit assignment problem is being solved here: Not only is learning in this task achieved by a highly time sensitive mechanism, such as an eligibility trace', but that this mechanism operates on a record of previous movements not a record of previous locations. The task in experiment 1 could be solved by a learning mechanism that stored target information, or trajectory information (since learning to move toward the target location, or moving in a target direction would both allow successful completion of the task). The target location method remains viable for experiment 2, but the task cannot be successfully completed by acquiring target trajectories—the start location of the movement shifts, requiring different trajectories to reach the target. The absence of learning in experiment 2, but successful learning in experiment 1, suggests that the participants are relying on a trajectory based strategy.

That a stable starting position could be so critical to learning is somewhat surprising. Previous work with an emphasis on spatial goals has shown that both rats and humans are capable of learning even when a stable trajectory is not associated with the goal (Tolman, 1948; Landau et al., 1984). Human visuo-spatial reasoning is highly developed and, for example, in tasks such as the pursuit rotor task (Frith and Lang, 1979) participants are able to trace a moving target so that current spatial position guides trajectory. In the Morris Water Maze (Morris, 1984) rats learn a target location rather than a trajectory or by using “dead reckoning” [but see Chamizo (2003)]. That our participants are not able to use spatial location to guide their movements suggests that our task taps a different set of processes. Indeed, we designed the task (Stafford et al., 2012) to rely as far as possible on the processes of motor learning without augmentation from visual-spatial memory or explicit reasoning. By using a task that tapped implicit motor processes we hoped to be able to isolate the specific capacities of this architecture of action discovery. Alternatively, it is also possible that the lack of reliable visuo-spatial information in this formulation of the task forces the system to rely on trajectory information to an unusual extent. Certainly the addition of spatial information in the form of a visual cue would have colored the results, and further experimentation is required to assess the relative contribution of each category of information on learning. However, what can be said with certainty is that in the absence of visuo-spatial information the system is capable of using only trajectory information from efference copy to learn spatial tasks.

If we consider how an animal might learn under natural conditions, it seems likely that the behavior it chooses to reselect—in effect, the unit of reinforcement—might relate to the attitude of its body and its overall position within the environment at the moment when reinforcement arrived. The ability to learn particular trajectories of movement might not be a key aspect of action acquisition because reinforcement is so rarely contingent on such movement. Indeed, there is mounting evidence to suggest that the motor output an animal is most inclined to reselect and reinforce might be its terminal body position rather than the movement trajectory required to achieve that body position. Graziano (2006) describes how attempts to map the motor cortex have revealed that actions do not appear to be represented at the neural level in the form of motor primitives that can be combined to form complete actions. Instead, particular portions of the cortex, when stimulated, evoke whole meaningful adaptive responses such as defensive or feeding postures. Furthermore, certain aspects of these actions appear to be more important than others. For example, hand movements are encoded in such a way that the hand will finish at a specific point in space, irrespective of where it started. Such representations do not describe a particular sequence of movements and instead describe behaviorally relevant terminal postures. In Graziano's view, certain features of actions, such as the final hand position, are crucial and the means by which these positions are achieved are of less importance and are likely free to vary to a greater extent. These representations in the cortical behavioral repertoire are plastic, and are able to represent complex movements as a function of experience and training (Martin et al., 2005; Ramanathan et al., 2006). While our current results may appear in tension with this body of evidence, one reconciliation is that regardless of the final representation in the cortex (which seemingly does include the terminal posture), a stable trajectory of movement is sufficient to support this process of learning. In other words, the conditions required for learning actions can be different from the eventual form of their storage.

These experiments validate the task as being a useful one for investigating the mechanisms of novel action learning (Stafford et al., 2012). The manipulation of delay allows us to expose the time sensitivity of these mechanisms (Walton et al., 2013), while precise stimulus control even allows us to discern the involvement of different neural pathways in action learning (Thirkettle et al., 2013). The current result suggests that trajectories can act as the substrate of novel action learning and further that in the absence of both visual information and a stable trajectory, actions can be discovered but cannot be refined over subsequent repetitions, although it should be noted that it remains possible that with more repetitions some improvement in performance could be observed.

We were inspired in this investigation by our theory of the function of the basal ganglia in novel action learning (Redgrave and Gurney, 2006; Redgrave et al., 2013). These variations of the “joystick task” are important and revealing as a whole because action acquisition presents a particularly difficult problem in the compromise between over-constrained and under-constrained tasks: when we over-constrain, we leave little opportunity for the agent to generate interesting behavioral variance as they freely explore and discover the new action; but when we under-constrain, there is simply too much noise in the data for us to draw any meaningful conclusions.

This work has been inspired by considering human action learning from the perspective of an autonomous agent which must acquire novel actions without either explicit instruction or certain knowledge of action-outcome relations (see also Shah et al., submitted). We have been guided in this by work in intrinsically motivated learning, and particularly by work within the framework of reinforcement learning (Sutton and Barto, 1998). From this reinforcement learning perspective a number of direct predictions flow. For example, the exploration-exploitation dilemma is a fundamental trade-off in learning within a complex space of actions where the reinforcement signal has unknown bounds. Early focus on actions with highest known value may lead to failure to discover the highest value actions in the long run, and—conversely—early exploration may lead to the discovery of the highest value actions in the long-term. In the joystick task this predicts that those participants who “explore” the movement space more in early trials—i.e., those who cover a greater distance reaching the target—will eventually settle on a more optimal path than those who explore less and “exploit” a sufficient path to the target. We have confirmed that this signature of an exploration-exploitation trade-off manifests in our joystick task (Stafford et al., 2012) as well as in at least one other domain of skill acquisition (Stafford and Dewar, 2013).

For an autonomous agent the credit assignment problem is deeply under-constrained—any aspect of the agent's behavior could potentially be causative of some novel outcome. The present experiment shows that the action learning system of human subjects have a bias to attribute cause to trajectory aspects of brief motor actions, rather than spatial aspects (resulting in the failure to learn seen in experiment 2). It is plausible to suggest that a “representational bias” may exist in these systems in order to narrow down their search of motor space for novel action-outcome pairs. An animal analog of the joystick task has been developed, and research already conducted demonstrates that general measures of behavior are comparable between rat and human participants (Stafford et al., 2012). Further work is required to assess whether the core challenge of the credit assignment problem is approached in a similar manner in rats, and whether search strategies and persistent elements of movement are detectable in rat response data and comparable to human data. Should this research reveal a common solution to the credit assignment problem, we would suggest that the use of a representational bias to focus the search of motor space could be a reasonable approach for any artificial agent.

The ability to acquire new actions is a keystone of human intelligence and the drive to explore our motor competency an essential element of our intrinsic motivations, relating, as it does, to aspects of intrinsic motivation such as novelty, surprise, curiosity, and mastery (Baldassarre and Mirolli, 2013). Our task allows us to frame general questions about intrinsic motivation and action discovery within a tightly controlled experimental context.

### Conflict of interest statement

The authors declare that the research was conducted in the absence of any commercial or financial relationships that could be construed as a potential conflict of interest.
